# Malnutrition-Related Liver Steatosis, CONUT Score and Poor Clinical Outcomes in an Internal Medicine Department

**DOI:** 10.3390/nu16121925

**Published:** 2024-06-18

**Authors:** Nicoletta Miano, Giorgia Todaro, Maurizio Di Marco, Sabrina Scilletta, Giosiana Bosco, Francesco Di Giacomo Barbagallo, Roberto Scicali, Salvatore Piro, Francesco Purrello, Antonino Di Pino

**Affiliations:** Department of Clinical and Experimental Medicine, University of Catania, 95122 Catania, Italy; nicoletta.miano@gmail.com (N.M.); giorgia.todaro92@gmail.com (G.T.); maurizio.dimarco@studium.unict.it (M.D.M.); sabrinascilletta@gmail.com (S.S.); giosiana.bosco@gmail.com (G.B.); fdigiacomobarbagallo@gmail.com (F.D.G.B.); roberto.scicali@unict.it (R.S.); salvatore.piro@unict.it (S.P.); francesco.purrello@unict.it (F.P.)

**Keywords:** malnutrition, hepatic steatosis, CONUT score, in-hospital outcomes, internal medicine

## Abstract

Fatty liver disease has been identified as a marker of malnutrition in different clinical settings. Recently, the COntrolling NUTritional status score (CONUT score) emerged as a promising tool for malnutrition assessment. Our aim was to evaluate short-term outcomes among patients with malnutrition-related liver steatosis in an Internal Medicine department. Furthermore, we evaluated the association of the CONUT score with malnutrition-related liver steatosis. Data from 247 patients hospitalized in an Internal Medicine department were retrospectively collected. The study population was stratified into three groups based on hepatic radiodensity assessed with computed tomography: mild steatosis (≥56.1 HU), moderate steatosis (between 49.7 and 56 HU), and severe steatosis (≤49.6 HU). We then calculated the CONUT score. Severe steatosis patients had higher in-hospital mortality (18.2 vs. 15.5%) and longer in-hospital stays compared with the mild steatosis group (length of in-hospital stay longer than 12 days: 45% vs. 40%). Logistic regression analysis showed that severe steatosis was not significantly associated with in-hospital all-cause death, while a high CONUT score was an independent risk factor for sepsis. We found an independent relationship between malnutrition-associated liver steatosis and the CONUT score. These results identified the CONUT score as a tool for nutritional assessment of hospitalized patients.

## 1. Introduction

Malnutrition (MN) is a condition developing from inadequate nutrient intake or absorption that results in altered body composition, reduced physical and mental function, and poor clinical outcomes [1,2].

One-third to one-half of patients hospitalized in medical or surgical departments suffer from protein–energy undernutrition, and the prognosis of the patients during hospitalization, in the short, medium, and long term, is significantly affected by nutrient deficiency, leading to worse outcomes [3,4].

The current evidence indicates that undernutrition can trigger the development of a type of liver steatosis different from conventional nonalcoholic fatty liver disease (NAFLD), which is basically related to overnutrition and obesity. This form of liver steatosis associated with malnutrition often arises in the context of an inadequate intake of essential nutrients, such as proteins, calories, and certain vitamins. The primary pathogenetic processes include deficiency in nutrients with hepatoprotective properties that support hepatic lipid metabolism, deficiency in chemicals required for the output of very low-density lipoprotein (VLDL), and modifications in the makeup and role of the intestinal microbiota [5,6,7]. It seems that malnutrition is linked to chronic liver disease, particularly in hospitalized patients, because of insufficient intake of macro- and micronutrients, which increases mortality and complications [8,9]. In fact, it has been found that a patient’s nutritional state might predict their prognosis if they have liver disease [10,11]. However, nutritional assessment is often neglected, and nutritional challenges in patients with liver steatosis and other chronic diseases are underestimated. Abdominal ultrasonography is the instrument of choice for hepatic examinations. Nevertheless, over the past 20 years, computed tomography (CT) has become more frequently used for the non-invasive evaluation of fatty liver disease, offering an accurate measurement of the liver’s fat content [12,13].

As concerns the diagnosis of malnutrition, there is not a validated gold standard approach yet, and the ones that are available are generally inadequate for routine clinical practice [14]. Accordingly, we evaluated the association of the CONUT score, a simple and easy-to-calculate clinical score, with malnutrition-related hepatic steatosis. The COntrolling NUTritional status score (CONUT score), which was recently introduced, seems to have a promising predictive impact in various clinical scenarios [15]. It was developed as an assessment tool for the early identification of low nutritional status and depends on the total peripheral lymphocyte count, serum albumin concentration, and levels of total cholesterol (TC). Protein reserves are shown by albumin; caloric depletion is shown by TC; and immunological defense is shown by lymphocyte count. An increased score paired with a lower level of nutrients is linked to a decline in each factor. The CONUT score was initially developed and evaluated in Surgical and Oncology departments to predict acute worsening during hospitalization. However, recently, it has also been analyzed in patients hospitalized in Internal Medicine departments, demonstrating that it has a significant prognostic impact for various clinical conditions such as chronic disease, cancer, and cardiac disorders [16,17,18]. The aim of this study was to evaluate short-term complications among patients with malnutrition-related liver steatosis admitted to an internal medicine department. Furthermore, given the clinical relevance of prompt recognition of patients with malnutrition, we evaluated the association of the CONUT score, a simple and easy way to calculate clinical scores with malnutrition-related hepatic steatosis.

## 2. Materials and Methods

### 2.1. Patients

We retrospectively acquired clinical and radiological data through the medical records of patients that underwent abdominal CT while admitted to the Internal Medicine and Geriatric departments of the Azienda Ospedaliera di Alta Specializzazione Garibaldi Nesima, Catania, Italy, from the months of September to December 2021. The data included the following: (1) age, gender, comorbidities (the presence of hypertension, diabetes mellitus, chronic heart failure, chronic kidney failure, neoplasm, previous stroke, chronic obstructive pulmonary disease (COPD), and chronic liver disease); (2) clinical occurrences during in-hospital stay (mortality, length of stay, diagnosis of sepsis, blood transfusions needed); (3) patients’ clinical and biochemical features at the time of admission, such as systolic and diastolic blood pressure, glycemia, creatinine, estimated glomerular filtration rate (eGFR), TC, high-density lipoprotein cholesterol (HDL-c), low-density lipoprotein cholesterol (LDL-c), triglycerides, total proteins, albumin, glutamic oxaloacetic transaminase, glutamic pyruvic transaminase, N-terminal fragment brain natriuretic peptide (NT-pro-BNP), procalcitonin, high-sensitivity C-reactive protein (hs-CRP), erythrocyte sedimentation rate (ESR), complete blood count, hemoglobin, hematocrit, and international normalized ratio (INR); (4) values of liver radiodensity on abdomen CT without contrast medium. We excluded all patients with a previous diagnosis of MAFLD in their clinical records, including those with alcoholic liver disease, autoimmune hepatitis, chronic liver disease of viral etiology, toxic damage associated with drugs, genetic accumulation of metals, or other genetically based liver diseases.

### 2.2. Evaluation of Liver Steatosis

Liver radiodensity was calculated on the basis of the X-ray attenuation, expressed in Hounsfield Units (HUs) and in agreement with the recent literature, by placing three circular regions of interest (ROIs) of 300 mm^2^ ± 10 mm^2^ in peripheral areas of the liver parenchyma, avoiding vessels, bile ducts, focal lesions, areas of parenchymal inhomogeneity or artifacts caused by the ribs or by the air present in the gastrointestinal tract: in particular, one ROI circle was positioned on the right anterior lobe, one on the right posterior lobe and one on the left lobe, on CT section where the right portal branch enters the liver; the mean of the three attenuation values, quantitative index of the liver fat content, was then calculated [12].

### 2.3. Calculations

The CONUT score was determined, in accordance with the first study results [15], from the serum albumin concentration, total peripheral lymphocyte count, and total cholesterol concentration. Albumin concentrations ≥ 3.5 g/dL, 3.0–3.49 g/dL, 2.5–2.99 g/dL, and ≤2.5 g/dL were scored as 0, 2, 4, and 6, respectively. Total lymphocyte counts ≥ 1600/mm^3^, 1200–1599/mm^3^, 800–1199/mm^3^, and ≤800/mm^3^ were scored as 0, 1, 2, and 3, respectively. Total cholesterol concentrations ≥ 180 mg/dL, 140–179 mg/dL, 100–139 mg/dL, and ≤100 mg/dL were scored as 0, 1, 2, and 3, respectively. The three scores were added together, resulting in the CONUT score. Patients were divided into two categories according to the degree of undernutrition as follows: low (0–4) and high (5–12) CONUT scores. The Chronic Kidney Disease Epidemiology Collaboration (CKD-EPI) formula was used to calculate the eGFR [19]. According to the original study, the Padua Prediction Score for the risk of venous thromboembolism was estimated [20]. We evaluated the high/low cut-offs of NT-proBNP, procalcitonin, AST, ALT, and high-sensitivity C-reactive protein according to upper laboratory limits as follows: NT-proBNP, 260 pg/mL; procalcitonin, 0.5 µg/L; AST, 34 UI/L; ALT, 55 UI/L; and hs-CRP, 0.5 mg/dL.

Our population was stratified into three equal-sized groups based on the values of hepatic radiodensity expressed in Hounsfield Units as an index of the hepatic fat content.

We identified two points within the data set: the first tertile (or lower tertile) and the second tertile (or upper tertile). These points corresponded to the following values: 56.1 and 49.7, so that one-third of the data lies below the first tertile and two-thirds below the second tertile. Thus, we defined three groups as follows: mild steatosis (≥56.1 HU), moderate steatosis (between 49.7 and 56 HU), and severe steatosis (≤49.6 HU). The three groups were composed as follows: 82 patients with mild steatosis, 82 patients with moderate steatosis, and 83 patients with severe steatosis.

### 2.4. Statistical Analysis

Statistical analyses of clinical and biological variables were conducted with Stat View 6.0 for Windows. The data are presented as median (IQR), means, or SD. The distributional properties of each variable, including normality, were evaluated using the Kolmogorov–Smirnov test. Group comparisons were conducted using ANOVA for continuous variables and the Chi-square test for non-continuous variables. We used a multinomial logistic regression model, setting the CONUT score as the independent variable and the group of steatosis as the dependent variable, to verify a possible association between the CONUT score and each category of steatosis. We applied logistic regression to investigate the independent association between fatty liver disease and clinical outcomes. We adjusted for the following variables: age, sex, cardiovascular disease, history of stroke, COPD, diabetes mellitus, chronic kidney disease, and tumors. A statistically significant finding was defined as a *p* value of less than 0.05. When required, logarithmic transformation was applied to numerical variables to lessen skewness; values are reported as the median and interquartile range.

### 2.5. Ethics

The Ethical Board Catania 2 approved this retrospective study (N° Protocol 370/CE; approval date of 21 May 2021). Every method performed in research projects involving human subjects complied with the Declaration of Helsinki and the ethical guidelines established by national and/or institutional research committees. Data collection was conducted retrospectively; thus, informed consent was not necessary.

## 3. Results

### 3.1. Baseline Characteristics, Medical History, and Comorbidities of the Patients

In total, data from 247 patients, 112 men and 135 women, were retrospectively collected based on clinical. Our population was separated into three groups according to the values of hepatic radiodensity expressed in HU as an index of the hepatic fat content: 82 patients with mild steatosis (≥56.1 HU), 82 patients with moderate steatosis (between 49.7 and 56 HU), and 83 patients with severe steatosis (≤49.6 HU).

The clinical and biochemical characteristics of the study population according to liver steatosis are shown in Table 1. The three groups were homogeneous for age, while a higher percentage of men was found in the mild steatosis group in comparison with those with severe steatosis (51.2% mild steatosis vs. 32.5% severe steatosis, *p* = 0.01). 

Patients in the severe steatosis group showed a significantly higher thromboembolic risk (4.2 ± 1.7 severe steatosis vs. 3.6 ± 1.8 mild steatosis; *p* = 0.16; 4.2 ± 1.7 severe steatosis vs. 3.3 ± 1.9 mild steatosis, *p* = 0.03). Moreover, patients with severe steatosis were more likely to have lower HDL cholesterol (25.7 ± 13.6 severe steatosis vs. 36.9 ± 15.5 mild steatosis; *p* < 0.0001) and albumin values (2.9 ± 06 severe steatosis vs. 3.2 ± 0.6 mild steatosis; *p* = 0.009), whereas they showed higher ESR values (68.4 ± 29.3 severe steatosis vs. 48.6 ± 30.5 mild steatosis; *p* = 0.007) and C-reactive protein (CRP) (97.5% severe steatosis vs. 80.5% mild steatosis; *p* = 0.0006). Furthermore, those with severe steatosis had higher values of white blood cells (WBC) (11.4 ± 10.2 severe steatosis vs. 9.4 ± 4.9 mild steatosis, *p* < 0.07; 11.4 ± 10.2 severe steatosis vs. 8.9 ± 4.1 mild steatosis, *p* < 0.02). Patients in the severe steatosis group showed higher INR values (1.4 ± 0.6 severe steatosis vs. 1.3 ± 0.2 mild steatosis; *p* < 0.09). Patients in the severe steatosis group had a higher CONUT score compared to those in the mild and moderate steatosis groups (6.2 ± 2.9 vs. 5.0 ± 3.0 *p* = 0.009, and 6.2 ± 2.9 vs. 5.9 ± 2.7 *p* < 0.0001, respectively).

Furthermore, we noted that the total bilirubin levels, as well as the levels of AST, ALT, and procalcitonin > 0.5 microg/L, increased from the mild steatosis group to the severe steatosis group, however, without statistical significance.

The medical history and comorbidities of the patients are shown in Table 2. Patients in the severe steatosis group were more frequently affected by oncological disease (40.5% severe steatosis vs. 24.7% mild steatosis, *p* = 0.03). Furthermore, patients in the severe steatosis group had a higher probability of having a stroke history, healthcare-related infections from multidrug-resistant germs, type 2 diabetes, neoplasms, and the need for blood transfusion during in-hospital stays, without statistical significance compared with those in the mild steatosis and moderate steatosis groups. 

### 3.2. Multinomial Logistic Regression Analysis to Assess the Association of CONUT Score to Each Steatosis Group

We performed a multinomial logistic regression using the CONUT score as the independent variable and the categories of steatosis (i.e., mild steatosis, moderate steatosis, and severe steatosis) as the dependent variable. We found that with an increasing CONUT score, the odds of being in the group with severe steatosis were significantly higher (OR 1.15, 95%CI 1.04–1.29, *p* = 0.01). However, the odds of being in the group with moderate steatosis were higher, but without reaching statistical significance (OR 1.11, 95%CI 0.99–1.24, *p* = 0.06).

### 3.3. Clinical Outcomes According to Steatosis Groups

In-hospital all-cause death occurred in 14 patients (18.2%) in the severe steatosis group vs. 11 patients (15.5%) in the mild steatosis group (Table 3). We showed that people with moderate steatosis were more likely to have sepsis compared to those in the mild steatosis group (42.1% moderate steatosis vs. 38.7% mild steatosis, *p* = 0.27).

The in-hospital stay was longer in the severe steatosis group compared with the mild steatosis group (length of in-hospital stay longer than 12 days: 45% severe steatosis vs. 40% mild steatosis).

### 3.4. Subgroup Analysis for the Primary Outcome Measure

We then performed logistic regression analysis and, after adjusting for confounders, found that severe steatosis was not significantly associated with in-hospital all-cause death [OR 0.94, 95%CI (0.87–1.01), *p* = 0.11]. Moreover, we demonstrated that a high CONUT score was an independent risk factor for sepsis [OR 1.34, CI 1.08–1.64), *p* = 0.005], while it was not significantly associated with in-hospital all-cause death [OR 1.26, 95%CI (0.96–1.63), *p* = 0.08].

To estimate the relationship between malnutrition-associated liver steatosis and the CONUT score, we performed multivariate logistic analysis, considering the mean liver density as a dependent variable and a number of clinical parameters as independent variables: age, sex, cardiovascular disease, history of stroke, chronic obstructive pulmonary disease, diabetes mellitus, chronic kidney disease, neoplasms, and CONUT score (Table 4). We found that mean liver density was inversely and independently related to the CONUT score (β = −0.26, *p* = 0.01).

## 4. Discussion

The aim of this study was to evaluate short-term in-hospital outcomes among patients with malnutrition related to liver steatosis admitted to an Internal Medicine Department. 

We found that severe steatosis was not significantly associated with in-hospital all-cause death after adjustment for multiple confounders. Most of the studies in the literature have focused on the role of a high-calorie diet in the pathogenesis of hepatic steatosis. Although this assumption is undoubtedly valid, in recent decades, numerous pathogenic mechanisms responsible for excessive lipid accumulation in the liver of undernourished subjects have been recognized. Indeed, it appears that fatty liver disease depends on many nutritional factors, and micronutrient deficiency (MND) seems to play a crucial role [5,6,7]. The association between liver disease and severe hypoalimentation was previously demonstrated in other clinical contexts. Hanachi M et al. demonstrated that a BMI < 12 was the sole independent risk factor for hepatic cytolysis in a study conducted in patients with anorexia nervosa [21]; furthermore, other studies have shown that increased caloric consumption and weight gain can lead to a rapid improvement in liver function tests [22]. Liver abnormalities have been reported as complications of other clinical conditions associated with impairment of nutrient absorption, such as bariatric surgery and intestinal failure [23,24]. These results support evidence from previous observations: chronic liver disease, ranging from steatosis (fatty liver) to steatohepatitis, acute alcohol-associated hepatitis, and liver cirrhosis, is associated with malnutrition, especially among hospitalized patients due to an inadequate intake of both macro- and micro-nutrients, leading to higher mortality and complications [8,9]. Currently, an unmet clinical need is the identification of a screening tool that could quickly identify patients who are more likely to have poorer clinical outcomes, primarily in the hospitalized population. Considering that patients admitted to Internal Medicine departments frequently suffer from malnutrition, it is crucial to find a simple score with a high predictive value to properly treat patients’ nutritional needs. Currently, several clinical tools have been proposed for nutritional evaluation; however, numerous difficulties have arisen in their clinical application. In this context, a straightforward, impartial measure of inflammation and nutritional status, which is gaining more reliability, is the CONUT score. The CONUT score is a comprehensive index that uses standard blood biochemical tests that are typically performed at admission for hospitalized patients in Internal Medicine units [15]. In a previous study, we demonstrated that patients hospitalized in an internal medicine department should be evaluated using the CONUT score to assess their nutritional status and, consequently, the risk of adverse outcomes due to malnutrition [16]. Furthermore, the utility of the CONUT score in identifying hospitalized malnourished patients with inadequate clinical results has been shown in other clinical settings. Our findings indicated that the CONUT score could be useful in assessing the nutritional state of patients admitted to an Internal Medicine ward. These results show that patients who are more prone to have negative in-hospital outcomes can be identified using the CONUT score as a nutritional screening tool. Moreover, this study highlighted an independent association between the CONUT score and malnutrition-related liver steatosis. To our knowledge, this is the first study in the literature to analyze this association. Indeed, previous studies explored the possible association between liver steatosis and malnutrition through different food frequency questionnaires. Petermann-Rocha et al. [25] showed that patients with higher scores at the 14-Item Mediterranean Diet Adherence Screener (MEDAS-14) and other scores, such as the Mediterranean Diet Score [26] and the Healthy Diet Indicator, strictly linked with better diet quality, had a significantly lower risk of severe liver steatosis compared with patients with worse nutrition habits. Furthermore, Matsui M. et al. [27] found that the prognostic nutritional index (PNI) was a predictor of nutritional status in patients with chronic liver disease, liver steatosis included. In particular, a PNI score of <40 was beneficial in predicting clinical outcomes for those suffering from long-term liver disease. 

People suffering from liver disease frequently experience malnourishment and are unable to have a proper oral food intake. Poor body composition and biological function can result from insufficient consumption and poor gastrointestinal absorption [25,27,28]. Nutritional status has been identified as a prognostic predictor for patients with liver disease [10,11]. Nevertheless, while treating patients with liver steatosis and other chronic diseases, nutritional evaluation is frequently overlooked, and nutritional problems in these patients are underestimated. Nutritional therapy interventions are therefore frequently underused for this group of patients [29]. This study has several strengths. Our study is the first, to our knowledge, to investigate the short-term prognostic value of the CONUT score in a cohort of patients with different grades of hepatic steatosis in an internal medicine department. Indeed, patients who are at risk for unfavorable outcomes and could benefit from nutritional supplementation could be easily identified thanks to the predictive value of a high CONUT score at admission. There are a few limitations that should be noted. Firstly, the study’s statistical power is limited due to the retrospective nature of this work and a small sample size. In addition, even though we adjusted for the measured confounders, other variables such as drugs and nosocomial infections were not taken into consideration. Third, we were unable to make any deductions regarding the patients’ long-term prognosis (i.e., re-hospitalization rate, death within the first month, loss of autonomy in daily living activities, etc.) since no information is currently given concerning the nutritional status or clinical outcomes of the patients after discharge. Finally, even if patients with a previous diagnosis of MAFLD in their clinical records were not included, we do not exclude that there may be an overlap between MAFLD and malnutrition-related liver steatosis, given the inclusive nature of the new definition of MAFLD.

## 5. Conclusions

The CONUT score is strictly associated with liver steatosis. Thus, the CONUT score can be used as a tool for nutritional assessment to recognize patients who require careful monitoring while hospitalized.

## Figures and Tables

**Table 1:** Clinical characteristics and blood test parameters at admission to an Internal Medicine department according to mean liver density tertiles.

	Mild Steatosis (*n* = 82)	Moderate Steatosis (*n* = 82)	Severe Steatosis (*n* = 83)
Age, years	71.9 ± 16.2	72.2 ± 14.4	71.9 ± 14.4
Sex, female %	51.2	45.1	27 *
Drugs in home therapy, *n*	6.2 ± 5.1	5.3 ± 3.9	6.2 ± 4.1
VTE risk (Padua Score)	3.6 ± 1.8	3.3 ± 1.9	4.2 ± 1.7 ^†^
SBP, mmHg	123.5 ± 18.3	126.2 ± 23.0	127.6 ± 18.8
DBP, mmHg	69.1± 11.8	70.7 ± 10.7	71.7± 10.0
CONUT score	5.0 ± 3.0	5.9 ± 2.7	6.2 ± 2.9 *^,†^
Mean liver density, HUs	61.5 ± 5.4	52.9 ± 1.8	43.6 ± 4.9
Fasting Glucose, mg/dL	111.1 ± 46.9	110.1± 46.6	105.4 ± 51.0
Urea mg/dL	62.0 ± 43.0	67.9 ± 59.1	62.5 ± 50.7
eGFR, mL/min/1.73 m^2^	68.8 ± 33.9	67.4 ± 31.3	70.1 ± 31.6
Albumin, g/dL	3.2 ± 0.6	3.0 ± 0.6 *	2.9 ± 0.6 *
Total bilirubin, mg/dL	0.9 ± 0.7	1.3± 2.3	1.6 ± 2.4
AST, UI/L	23.5 (19–37)	20 (15–31)	27 (19–47) ^†^
ALT, UI/L	15 (10–37)	14 (6–28)	18 (8–29)
GGT UI/L	29 (18–44)	37 (18–77)	34 (20–111)
ALP UI/L	70 (58.2–86)	74.5 (60–115)	74 (61–122)
NT-proBNP > 260 pg/mL, %	35.3	18.2	13.5
hs-CRP > 0.5 mg/dL, %	80.5	88.5	79 *^,†^
Procalcitonin > 0.5 µg/L, %	23.5	26.0	32.4
WBC, 10^3^/µL	9.4 ± 4.9	8.9 ± 4.1	11.4 ± 10.2
Neutrophils, 10^3^/µL	7.1 ± 4.7	6.4 ± 3.8	8.7 ± 8.6
Lymphocites, 10^3^/µL	1.5 ± 1.6	1.4 ± 0.8	1.5 ± 1.3
Platelets, 10^3^/µL	249.5 ± 122.5	225.8 ± 113.0	227.9 ± 119.5
HB, g/dL	11.4 ± 2.7	10.7 ± 1.8	10.9 ± 2.2
INR	1.3 ± 0.2	1.3 ± 0.2	1.4 ± 06
Total cholesterol, mg/dL	150.9 ± 42.1	147.5 ± 52.3	140.3 ± 59.3
LDL cholesterol, mg/dL	89.5 ± 36.6	91.2 ± 43.2	87.3 ± 55.8
HDL cholesterol, mg/dL	36.9 ± 15.5	32.8 ± 13.6 *	25.7 ± 13.6 *^,†^
Triglycerides, mg/dL	113 (81–142)	118 (92–154)	111 (78–159)

Data are presented as percentage, mean ± SD, or median (IQR). VTE: venous thromboembolism; SBP: systolic blood pressure; DBP: diastolic blood pressure; CONUT: controlling nutritional status; HUs: Hounsfield Units; eGFR: estimated glomerular filtration rate; AST: aspartate aminotransferase; ALT: alanine aminotransferase; GGT: Gamma Glutamyl Transpeptidase; ALP: Alkaline Phosphatase; NT-proBNP: N-terminal pro-brain natriuretic peptide; hs-CRP: high-sensitivity C-reactive protein; WBC: white blood cells; HB: hemoglobin; INR: international normalized ratio; LDL: low-density lipoprotein; HDL: high-density lipoprotein. * *p* < 0.05 vs. mild steatosis; ^†^ *p* < 0.05 vs. moderate steatosis.

**Table 2:** Comorbidities according to mean liver density tertiles.

	Mild Steatosis (*n* = 82)	Moderate Steatosis (*n* = 82)	Severe Steatosis (*n* = 83)
Cardiovascular disease, %	67.9	75	64.5
History of stroke, %	2.7	4.6	11.3
COPD, %	27.5	29.1	28.8
Diabetes mellitus, %	29.6	26.6	32.5
CKD, %	21.0	25.6	22.7
Neoplasms, %	24.7	36.6	40.5 *
MDR germs isolation, %	15.8	15.6	20.4
Patient needing blood transfusions, %	15.8	15.6	27.3

Data are presented as percentage. COPD: chronic obstructive pulmonary disease; CKD: chronic kidney disease; MDR: multidrug resistant; * *p* < 0.05 vs. mild steatosis.

**Table 3:** In-hospital outcomes according to mean liver density tertiles.

	Mild Steatosis (*n* = 82)	Moderate Steatosis (*n* = 82)	Severe Steatosis (*n* = 83)
In-hospital mortality, %	15.5	17.7	18.2
Length of in-hospital stay >12 days, %	45.5	43	44.6
Diagnosis of sepsis, %	38.7	42.1	31.6

Data are presented as percentage.

**Table 4:** Multiple regression analysis evaluating major determinants of mean liver density.

Independent Variables	Coefficient β	*p*
Age	−0.07	n.s.
Male sex	−0.08	n.s.
Cardiovascular disease	0.09	n.s.
History of stroke	−0.19	0.03
COPD	0.12	n.s.
Diabetes mellitus	−0.13	n.s.
CKD	0.04	n.s.
Neoplasms	−0.07	n.s.
CONUT score	−0.26	0.01

COPD: Chronic obstructive pulmonary disease; CKD: chronic kidney disease; CONUT: controlling nutritional status; n.s.: not significant.

## Data Availability

Data from this study are available upon request from corresponding author. Data are not publicly available due to privacy.
